# Wild imitating vs greenhouse cultivated *Dendrobium huoshanense*: Chemical quality differences

**DOI:** 10.1371/journal.pone.0291376

**Published:** 2024-01-25

**Authors:** Li Hu, Shiwen Wang, Lin Zhang, Liangliang Shang, Ruiye Zong, Jinyan Li, Zhanghua Wu, Yuanjun Meng, Yafeng Dai, Yuechun Huang, Gang Wei

**Affiliations:** 1 School of Pharmaceutical Science, Guangzhou University of Chinese Medicine, Guangzhou, China; 2 Jiuxianzun Dendrobium Huoshanense Co. Ltd., Lu’an, China; 3 The First School of Clinical Medicine, Guangzhou University of Chinese Medicine, Guangzhou, China; 4 The First Affiliated Hospital, Guangzhou University of Chinese Medicine, Guangzhou, China; University of Kotli, PAKISTAN

## Abstract

*Dendrobium huoshanense* (*D*. *huoshanense*) has been used as functional food supplements and herbal medicines for preventing and managing diseases with a long history in China. Due to its endangered natural resources and huge demand, people tend to cultivate *D*. *huoshanense* to protect this species. However, the quality of wild and cultivated herbs of the same species may change. This work quantified and compared the main quality traits and chemical components of wild imitating and greenhouse cultivated *D*. *huoshanense* with different growth years. As a result, wild and cultivated *D*. *huoshanense* had similar chemical composition, but there are significant differences in the content of many ingredients (polysaccharides, flavonoids, nucleosides, bibenzyls, lignans and volatile compounds). And the contents of many of these components increased with growing years. In addition, multivariate statistical analyses have been applied to classify and evaluate samples from different cultivation modes according to these components. In conclusion, our results demonstrated that the overall quality of greenhouse cultivated *D*. *huoshanense* was not as good as wild-grown, but this mode can be a promising and sustainable way of producing *D*. *huoshanense*.

## Introduction

*Dendrobium* is one of the three largest genera in the family Orchidaceae, with over 1,000 species worldwide [1, 2]. In China, there are 74 species and 2 varieties, more than 50 of which can be used medicinally [3, 4]. *Dendrobium huoshanense* (*D*. *huoshanense*), locally known as “Mihu”, is the most precious species of *Dendrobium*. The stems of *D*. *huoshanense* has been used as a food material to make soup and porridge and a functional medicine to nourish the stomach, relieve throat inflammation and promote body fluid production for thousands of years in Chinese medicine [5, 6]. Furthermore, their stems can be twisted into a spring or spiral shape, heated and dried to prepare “Fengdou”, which used as flavorful teas and nutritional supplements [7]. Modern research has shown that *D*. *huoshanense* contains diverse compositions, mainly including polysaccharides, flavonoids, bibenzyls, lignans, nucleotides, volatile compounds (VOCs), amino acids, alkaloid, etc [5, 8], and possesses various biological functions, mainly including immunomodulatory [9], anti-inflammatory [10], antitumor [11], antioxidative [12], hepatoprotective activities, etc [13]. These active ingredients are used singly or in combinations to make *D*. *huoshanense* of high medicinal value.

*D*. *huoshanense* is mainly produced in Ta-pieh Mountain area, Huoshan County, Anhui Province (China), with very narrow geographical distribution area [14]. In the past, people have been using *D*. *huoshanense* from the wild. Nowadays, the existing wild resources has been endangered due to excessive collection as well as the extremely low germination rate of natural *Dendrobium* seeds. *D*. *huoshanense* has been listed in the list of national key protected wild plants as a class I protected species [15]. To protect wild resources, in recent years, the development of tissue culture and cultivation technology has greatly expanded its planting area, alleviating the shortage of resources and other problems to a certain extent. According to statistics, as of 2021, the planting area of *D*. *huoshanense* was about 700 hm^2^, most of which were in Huoshan County [16]. This included the development of imitated wild and greenhouse cultivation modes. Under wild imitating cultivation mode, the tissue culture seedlings of *D*. *huoshanense* were fixed on granite with better water absorption in mountain forest, allowing it to grow naturally. This pattern is closest to the wild growing environment of plants. The greenhouse cultivation mode is a kind of facility cultivation mode with bark as the substrate. This mode refers to the creation of artificially controlled environmental conditions that allow plants to grow and develop normally. However, the chemical compositions may vary depending on its cultivation environment. According to studies, many external environmental factors such as light, water, temperature will influence the accumulation of secondary metabolites in plants [17–20]. Furthermore, growth years are also important factor to affect the accumulation of secondary metabolites [21–23].

Currently, *D*. *huoshanense* has attracted increasing attention about its biochemical properties and functions, as well as its commercial applications in food, nutrition and medicine. In 2019, local standards for *D*. *huoshanense* (stems by artificial cultivation) as a food were officially promulgated. It was associated by the general public that wild imitating cultivated *D*. *huoshanense* had improved nutritional properties and higher value than greenhouse cultivated *D*. *huoshanense*. However, there is a need for better scientific data to prove. Moreover, locally, the stems of less than two years old could be harvested for food. The question arises whether the stems with less than 2-year-old contain enough health-promoting compounds compared to those more than 2-year-old stems. Nevertheless, there is a lack of systematic research on the effects of cultivation mode and growth years on the chemical ingredients to reveal the differences between them. And most relevant studies seemed to focus on the total accumulation of chemicals while individual compounds were ignored.

Therefore, the aim of this study is to investigate comprehensively the phytochemical and nutritional differences of different cultivation mode and growth years of *D*. *huoshanense*, including polysaccharides, nucleosides, flavonoids, bibenzyls, lignans, and volatile compounds through high performance liquid chromatography (HPLC), gas chromatography-mass spectrometry (GC-MS) and chemometric statistical methods. These results could provide an effective reference basis for identification and quality evaluation of *D*. *huoshanense* derived from the two different cultivation modes, and provide a theoretical basis for its development on functional foods and therapeutic agents.

## Materials and methods

### Materials and chemicals

**Fig 1:**
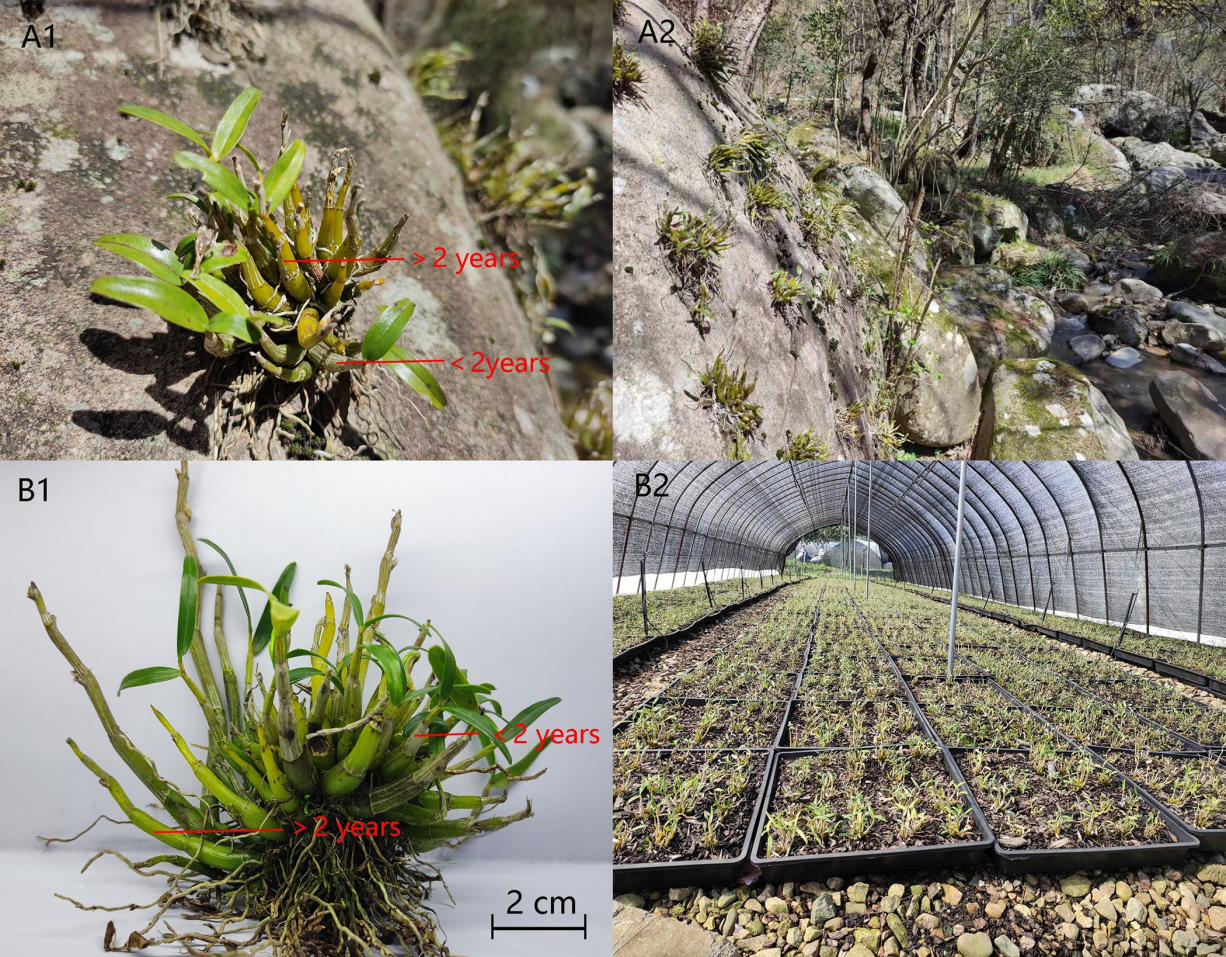
Phenotype and external growth environments of wild imitating (**A1 & A2**) and greenhouse (**B1 & B2**) *D*. *huoshanense*.

A total of 28 baches of *D*. *huoshanense*, including 14 batches of wild imitating cultivation and 14 batches of greenhouse cultivation samples, were all collected at Huoshan County of Anhui Province (116.317°E, 31.383°N) in March 2021. Based on the date of cultivation and combined with the appearance of characteristics, the samples from same tuft were divided into two different growth years (more than 2 years & less than 2 years) (Table 1), their phenotype and external growth environments were shown in Fig 1. All stems were cut into small pieces, dried in an oven at 60°C, ground into a powder and passed through a 65-mesh (0.25 mm) sieve.

**Table 1:** The samples information of *D*. *huoshanense* with two cultivation modes.

Wild imitating cultivation mode		Greenhouse cultivation mode	
Samples number	Growth years	Samples number	Growth years
W1-1	<2 years	G1-1	<2 years
W1-2	>2 years	G1-2	>2 years
W2-1	<2 years	G2-1	<2 years
W2-2	>2 years	G2-2	>2 years
W3-1	<2 years	G3-1	<2 years
W3-2	>2 years	G3-2	>2 years
W4-1	<2 years	G4-1	<2 years
W4-2	>2 years	G4-2	>2 years
W5-1	<2 years	G5-1	<2 years
W5-2	>2 years	G5-2	>2 years
W6-1	<2 years	G6-1	<2 years
W6-2	>2 years	G6-2	>2 years
W7-1	<2 years	G7-1	<2 years
W7-2	>2 years	G7-2	>2 years

The standards including D-mannose (purity ≥ 99.6%), D-glucose (purity ≥ 99.9%), uridine (purity ≥ 99.6%), guanosine (purity ≥ 93.6%), adenosine (purity ≥ 99.7%), schaftoside (purity ≥ 98%), gigantol (for identification purposes) were purchased from China National Institute for Food and Drug Control (Beijing, China), isoschaftoside (purity ≥ 98%) were purchased from Extrasynthese Chemical S.A.S (Lyon, France), and syringaresinol (purity ≥ 98%), syringaresinol-4-O-β-D-glucopyranoside (purity ≥ 98%), syringaresinol-4,4-O-β-D-di-glucopyranoside (purity ≥ 98%) were purchased from Wuhan Hengbiao Experimental Technology Co., Ltd (Wuhan, China). Besides, internal standard substance glucosamine hydrochloride (purity > 98%) was from Chengdu Ruifansi Biological Technology Co., Ltd (Chengdu, China).

Acetonitrile (ACN) and methanol (MeOH) were HPLC grade regents from Merck (Darmstadt, Germany). Ultrapure water was prepared using a Milli-Q water purification system (Billerica, MA, USA). Other reagents were of analytical grade.

### Sample preparation and analysis conditions

#### Determination of polysaccharides content

HPLC with pre-column derivatization has been widely used for qualitative and quantitative analyses of monosaccharides in plants polysaccharides [24]. Determination of polysaccharide content was carried out using Agilent 1260 system (Agilent Technologies, USA) equipped with a diode array detector (DAD) and Kromasil 100–5 C18 column (250 mm × 4.6 mm, 5 μm). The extraction of samples and analysis conditions were conducted according to Wu et al. [25].

Sample solution: Take the sample powder about 0.12 g, weigh it accurately, and place it in a Soxhlet extractor. Added 80% ethanol and heated for reflux extraction for 4 h. Ethanol was discarded and ethanol in the powder was dried. Disassemble the filter paper tube and place it in a beaker, add 100 mL water, then add 2 mL internal standard solution precisely, boil for 1 h and stir constantly. Cool the decoction, add water to about 100 mL, mix well, centrifuge, and set aside. Absorb 1 mL of the above sample solution and put into ampoule or head space bottle. Then, add 0.5 mL of 3.0 mol/L hydrochloric acid solution, sealed, mixed, hydrolyzed for 60 min at 110°C. Cooled the solution, and adjusted pH to neutral with 3.0 mol/L sodium hydroxide solution for later use. Absorb 400 μL of the above two solutions, add 0.5 mol/L PMP methanol solution, 0.3 mol/L sodium hydroxide solution 400 μL, mix, and react in water bath at 70°C for 100 min. Then add 0.3 mol/L hydrochloric acid solution 500 μL, mix well, wash with chloroform three times, 2 mL each time. Discard the lower layer of trichloromethane and retain the upper aqueous solution. The solution was filtered through a 0.45 μm membrane filter before HPLC analysis.

Analysis conditions: Agilent 1200 system (Agilent Technologies, USA) equipped with a diode array detector (DAD); chromatographic column: Kromasil 100–5 C18 column (250 mm × 4.6 mm, 5 μm); mobile phase: acetonitrile (A), and 0.02 mol/L acetonitrile (B); gradient elution program: 0 ~ 5 min, 16% A; 5 ~ 17min, 16% ~ 19% A; 17 ~ 30 min, 19% ~ 22% A, 30 ~ 35 min, 22% ~ 16% A; flow rate: 1.0 mL/min; temperature: 40°C; injection volume: 10 μL; detection wavelength: 250 nm.

#### Determination of nucleosides content

Determination of nucleoside content were analyzed with Agilent 1260 system (Agilent Technologies, USA) equipped with a diode array detector (DAD) and Zorbax SB Aq column (250 × 4.6 mm, 5 μm). Through the optimization of extraction and chromatographic conditions, the method was determined as follows:

Sample solution: Accurately weighed 0.2 g amount of *D*. *huoshanense* powder and placed it into a conical flask. Then added 20 mL 80% methanol, ultrasonic extraction for 1 hour at room temperature. Filtered the extracted solution with filter paper and concentrated them to dryness. Dissolve the residue with 60% methanol into 2 mL volumetric bottle and shake well. After 0.45 μm microporous filter membrane, the sample solution is obtained.

HPLC conditions: methanol (A) and water (B); gradient elution program: 0 ~ 10 min, 1% ~ 5% A; 10 ~ 15 min, 5% ~ 15% A; 15 ~ 20 min, 15% ~ 20% A; 20 ~ 30 min, 20% A; 30 ~ 35 min, 20% ~ 1% A; flow rate: 1.2 mL/min; temperature: 20°C; injection volume: 4 μL; detection wavelength: 260 nm.

#### Fingerprinting analysis of flavonoids, bibenzyls and lignans and content determination

The HPLC characteristic chromatogram analysis and content determination of flavonoids were determined according to the method established by our group previously [26].

Sample solution: Accurately weighed 1.0 g amount of *D*. *huoshanense* powder and placed it into a conical flask. Then added 50 mL methanol, ultrasonic extraction for 1 hour at room temperature. Filtered the extracted solution with filter paper and concentrated them to dryness. Dissolve the residue with methanol into a 5 mL volumetric bottle and shake well. After 0.45 μm microporous filter membrane, the sample solution for HPLC analysis is obtained.

Analysis conditions: Agilent 1200 system (Agilent Technologies, USA) equipped with a diode array detector (DAD); chromatographic column: Kromasil 100–5 C18 column (250 mm × 4.6 mm, 5 μm); mobile phase: acetonitrile and methanol (v/v = 1:1) (A) and 0.01 mol/L acetonitrile (B); gradient profile: 0 ~ 20 min, 14% ~18% A; 20 ~ 35 min, 18% ~ 22% A; 35 ~ 45 min, 22% ~ 26% A; 45 ~ 55 min, 26% ~ 30% A; 55 ~ 65 min, 30% ~ 36% A; 65 ~ 75 min, 36% ~ 40% A, 75 ~ 90 min, 40% ~ 60% A; 90 ~ 100 min, 60% ~ 70% A; 100 ~ 110 min, 70% ~ 80% A; 110 ~ 115 min, 80% ~ 14% A; flow rate: 0.8 mL/min; temperature: 40°C; injection volume: 10 μL; The UV detector was set at 340 nm for flavonoids and 215 nm for bibenzyls and lignans with full spectral scanning from 190 to 400 nm. The contents of two flavonoids and three lignans were determined simultaneously.

#### Analysis of volatile compounds

Through the optimization of sample extraction conditions and chromatographic conditions, the method was determined as follows: Sample solution: Accurately weighed 1.0 g amount of sample powder and placed it into a round-bottom flask. Then added 50 mL ethyl acetate, heated reflux extraction for 2 hours at 77°C. Filtered the extracted solution with filter paper and concentrated them to dryness. Dissolve the residue with ethyl acetate into a 1 mL volumetric bottle and shake well. After 0.22 μm microporous filter membrane, the sample solution for GC-MS analysis is obtained. GC conditions: the carrier gas is high purity helium (with purity of 99.999%). The programmed temperature condition was an initial temperature of 50°C, maintained for 3 min, then increased to 270°C at 10°C/min, maintained for 20 min, total analysis time was 45 min. The split ratio was 2: 1; gas flow rate: 1.2 mL/min; injection volume: 1 μL. MS conditions: ions were generated by a 70eV electron with an electron impact (EI) ionization mass spectrometric detector (MSD). The ion source and quadrupole temperature were 230°C and 150°C, respectively. Quadrupole mass spectrometry was performed using the full-scan method from 20 to 550 (m/z). The solvent delay was 3 min.

### Statistical analysis

Construction and comparison of HPLC characteristic chromatogram was conducted by the similarity evaluation system for chromatographic fingerprint of traditional Chinese medicine 2004A (Chinese Pharmacopoeia Commission). The contrast chromatogram was generated by means method and the peak areas in the integrated fingerprint were used for relative quantitative analysis. Differences in chemical components of *D*. *huoshanense* with two cultivation modes and two growth years were evaluated by principal component analysis (PCA), orthogonal partial least-squares discriminant analysis (OPLS-DA), variable importance in the projection (VIP), and one-way analysis of variance (ANOVA), and differences were considered statistically significant at *P* < 0.05 or *P* < 0.01 (Statistical Product and Service Solutions (SPSS) version 25, USA).

## Results and discussion

### Method validation of polysaccharides, nucleosides, flavonoids and lignans content determination

Data in Table 2 indicated that the five compounds expressed reliable regression equations and good linearity with r^2^ above 0.999 in their wide concentration ranges. The RSD values for method precision, repeatability, and sample stability of the seven analytes were all <3.0%. The overall recoveries were ranged from 97.11% to 103.99% with RSD<3%. Hence, this method is accurate, precise, and sensitive to conduct content determination of the polysaccharides and nucleosides.

**Table 2:** Regression equation, linearity range, correlation coefficient, and average recovery of 10 investigated compounds of *D*. *huoshanense*.

Compounds	Regression equation	Linearity range (μg/mL)	r^2^	Precision (RSD, %) (n = 6)	Repeatability (RSD, %) (n = 6)	Stability (RSD, %) (n = 6)	Recovery (%) (n = 6)	
							Mean	RSD
Mannose	Y = 1.0036X-0.0380	0.3684~29.96	0.9997	0.73	0.87	1.20	97.11	2.18
Glucose	Y = 0.7928X+0.1724	0.3750~30.00	0.9997	1.47	1.36	1.76	97.52	2.79
Schaftoside	Y = 1739.9X-0.3859	0.0519~1.0380	0.9999	2.27	2.64	2.87	99.56	2.21
Isoschaftoside	Y = 1832.5X-2.2940	0.0428~0.8560	0.9999	1.83	2.87	2.59	98.29	3.02
Uridine	Y = 1909.4X+0.4194	0.0084~0.2520	1.0000	0.32	2.77	0.57	102.08	1.92
Guanosine	Y = 1616.2X+0.6969	0.0096~0.2880	1.0000	0.51	2.87	2.86	99.79	2.00
Adenosine	Y = 2601.2X+0.4327	0.0092~0.2760	1.0000	0.69	2.21	1.39	100.39	2.28
Syringaresinol-di-glucopyranoside	Y = 3033.7X+30.52	0.03402~1.3606	0.9993	0.97	1.88	2.63	97.87	2.21
Syringaresinol-glucopyranoside	Y = 6269.2X+26.82	0.05101~2.0404	0.9999	0.50	2.52	0.67	103.99	0.70
Syringaresinol	Y = 9405.4X-11.21	0.02015~0.8060	1.0000	2.43	2.57	2.12	99.71	2.89

### Chemical composition

#### Polysaccharides composition and monosaccharide ratio

Polysaccharides were major medicinally active ingredients in *D*. *huoshanense*, which were used to assess the quality of *D*. *huoshanense* in Pharmacopoeia of the People’s Republic of China (2020) [27]. The contents of the total polysaccharide and the main monosaccharides are the essential quality assessment criterion for *Dendrobium*. Due to the differences in origins, regions of production, and cultivation conditions, the structure, quality and functions of plant polysaccharides can vary significantly [28]. Previous study has shown that the polysaccharides of *D*. *huoshanense* mainly consist of two monosaccharides, D-mannose and D-glucose [29, 30]. Therefore, it is more feasible to replace the total polysaccharide content with the sum of these two monosaccharides. Quantification in this study was based on an internal standard method using calibration curves fitted by linear regression analysis. The validated HPLC method was subsequently applied to the determination of 28 batches of *D*. *huoshanense*, and the quantitative analyses of mannose and glucose were summarized in Table 3. As the results shown, there were significant differences in polysaccharide content among the samples with different growth years and cultivation methods. *D*. *huoshanense* cultivated in greenhouse showed overall higher total polysaccharide content (mean of two different growth years was 36.23% and 22.44% respectively) than that of imitation wild (mean of two different growth years was 26.24% and 12.87% respectively). In addition, it is obvious that samples with more than 2-year-old growth had lower polysaccharide content in both cultivation environments. Particularly, many imitation wild samples with more than 2-year-old had very low levels (Detailed results were shown in S1 Table). Pharmacopoeia of the People’s Republic of China (2020) stipulates that the polysaccharide content of *D*. *huoshanense* should not be less than 15%. Our results showed that the polysaccharide content of greenhouse cultivation mode was far beyond the standard. However, many wild imitation samples with growth years of more than 2 years do not meet the standard. It also suggested that polysaccharide content should not be the only criterion for quality evaluation of imitation wild *D*. *huoshanense*.

**Table 3:** Contents of 10 compounds and monosaccharide ratio of wild imitating (W) and greenhouse cultivated (G) *D*. *huoshannense* with different growth years (mean ± SD).

Samples	Polysaccharide Content (%)				Nucleoside Content (μg/g)			Flavonoid Content (μg/g)		Lignans Content (μg/g)		
	D-mannose	D-glucose	TPC	Am/Ag	Uridine	Guanosine	Adenosine	Schaftoside	Isoschaftoside	Syringaresinol-di-glucopyranoside	Syringaresinol-glucopyranoside	Syringaresinol
W2^-^	12.05±2.66	14.29±3.06	26.34±5.35	0.92±0.16	215.99±41.67	247.87±64.97	275.36±32.36	62.92±19.47	89.81±42.92	128.38±30.08	358.39±125.29	53.12±19.61
W2^+^	5.16±3.49	7.71±2.24	12.87±5.62	0.65±0.36	205.00±27.80	218.50±35.93	234.94±16.99	68.86±24.46	129.20±56.11	304.82±62.89	442.12±195.65	99.69±41.56
G2^-^	21.29±2.79	14.95±1.62	36.23±2.07	1.58±0.35	159.56±20.26	186.20±27.59	175.42±34.96	81.45±31.02	123.90±47.11	96.06±25.35	285.50±70.11	42.15±16.17
G2^+^	12.69±2.83	9.75±3.21	22.44±5.41	1.49±0.41	166.02±30.44	188.82±38.49	189.94±33.66	64.67±26.14	121.52±54.11	269.85±49.62	378.45±87.05	62.28±22.12
*P*_*1*_	<0.01	>0.05	<0.01	<0.01	<0.01	<0.05	<0.01	>0.05	>0.05	>0.05	<0.01	>0.05
*P*_*2*_	<0.01	<0.01	<0.01	<0.01	>0.05	>0.05	<0.05	>0.05	>0.05	<0.01	>0.05	<0.05
*P*_*3*_	<0.01	<0.01	<0.01	<0.01	>0.05	>0.05	>0.05	>0.05	>0.05	<0.01	<0.05	<0.05

2^-^: growth years with less than 2 years; 2^+^: growth years with more than 2 years; TPC: Total polysaccharide content; Am/Ag: monosaccharide ratio; *P*_*1*_: significant difference between wild imitating and greenhouse cultivation mode; *P*_*2*_: significant difference between two different growth years of wild imitating samples; *P*_*3*_: significant difference between two different growth years of greenhouse cultivation samples.

Meanwhile, the peak area ratio of mannose to glucose was analyzed. The ratio range of imitating wild samples with different growth years was 0.92 and 0.65, while the greenhouse samples was 1.58 and 1.49 (Table 3). Obviously, there was a great difference in the ratio of mannose to glucose between these two cultivation modes, among which imitation wild is basically below 1 and greenhouse is above 1. Studies have shown that the compositional ratio of mannose to glucose may be an important factor in the expression of immunostimulatory activity [31]. Actually, in previous study, we have found that the content of mannose related in polysaccharides from imitating wild *D*. *officinale* was significantly higher than that from greenhouse plants [32]. However, it was opposite in *D*. *huoshanense*. As a result, the ratio of mannose and glucose of *D*. *huoshanense* might be used as the reference for the identification of these two cultivation methods.

#### Nucleosides composition

**Fig 2:**
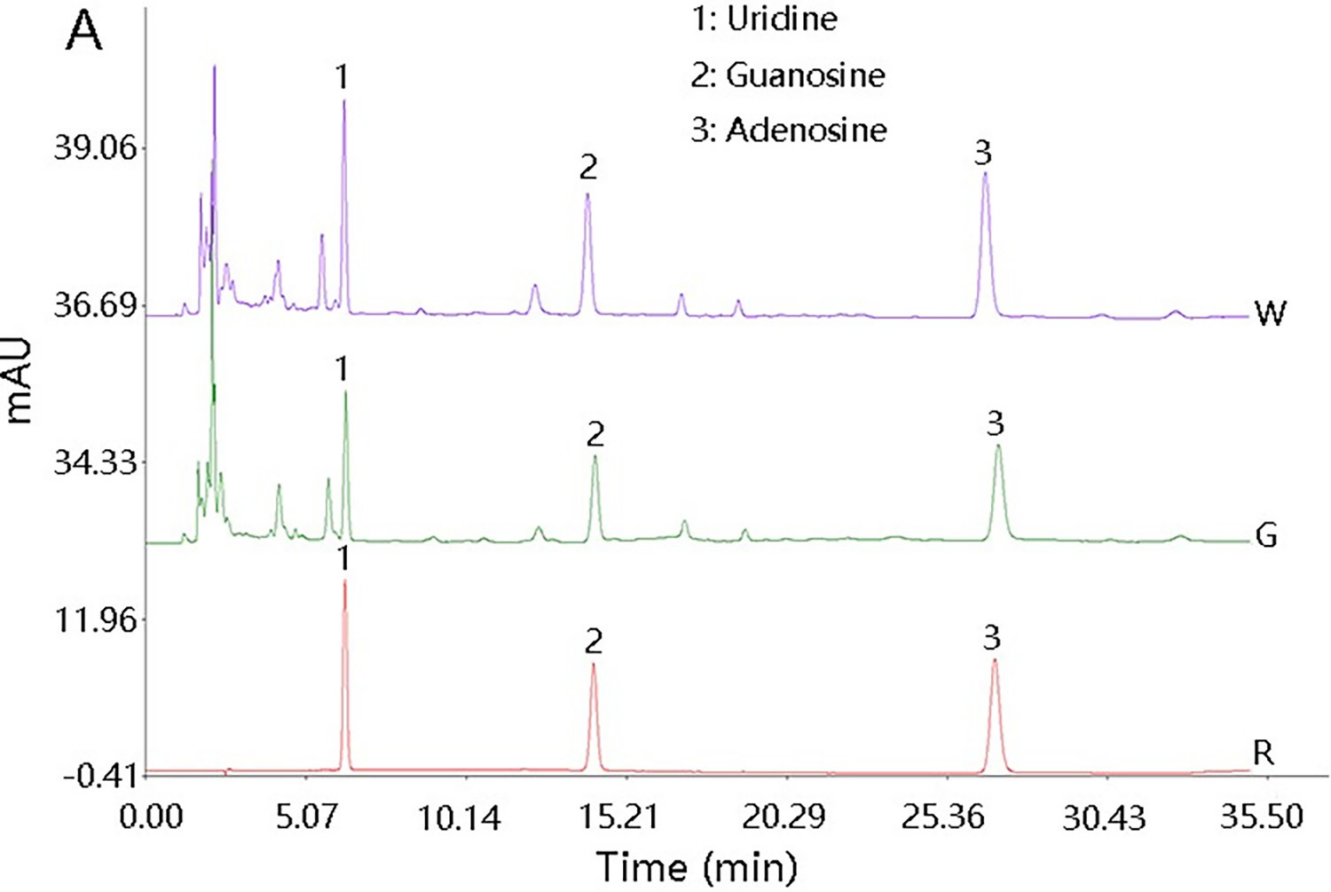
Chromatogram of the three nucleosides of wild imitating (W), greenhouse (G) *D*. *huoshanense* and the reference substance (R) by HPLC.

*Dendrobium* contains a variety of nucleosides, among which *D*. *huoshanense* and *D*. *officinale* have the highest contents and previous studies used HPLC-MS for qualitative and quantitative analysis of nucleosides [33, 34]. In our study, the established HPLC method was more rapid and convenient to detect the content of uridine, guanosine and adenosine of *D*. *huoshanense*. The kind of nucleoside components of *D*. *huoshanense* were consistent between two cultivation methods, but there were some differences in the content (Fig 2 and Table 3). Wild imitating cultivated *D*. *huoshanense* (the mean ranges from 205.00 to 275.36 μg/g) had higher nucleoside content than that of greenhouse (the mean ranges from 159.56 to 189.94 μg/g) (Detailed results were shown in S1 Table).

### Flavonoids, bibenzyls and lignans composition

Currently, chromatographic fingerprints can provide rich information of complex chemical constituents, which is one of the important means of quality control of Chinese herbal medicines, including the identification and discrimination of some closely related species [35, 36]. HPLC based fingerprinting method has good applicability in authenticity and quality evaluation. Our group established the fingerprint of flavonoids of *D*. *huoshanense* cultivated in greenhouses in the early stage [26], but the fingerprint of multiple components of the wild grown has not been analyzed, and whether it has the same characteristic peak as that cultivated in greenhouses is still unknown. Here, we analyzed the fingerprint chromatogram of flavonoids, bibenzyls and lignans composition at two wavelengths.

**Fig 3:**
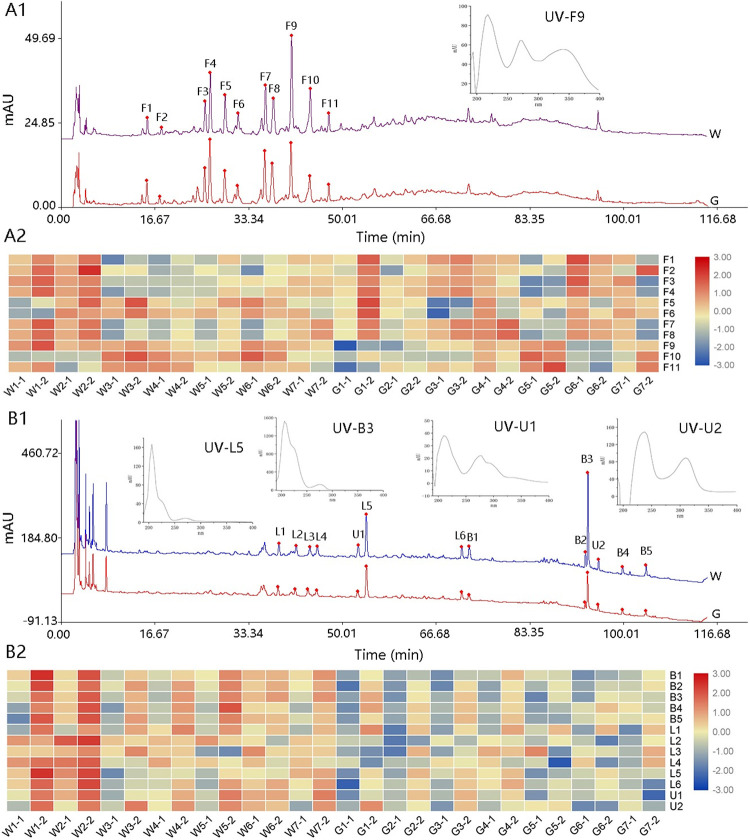
The contrast chromatograms, common characteristic peaks, UV spectrum of the corresponding compound and heat map of peak area of wild imitating (W) and greenhouse (G) *D*. *huoshanense* at 340 nm (**A1 & A2**) and 215 nm (**B1 & B2**).

At 340 nm, the best wavelength for flavonoids detection, 11 common characteristic peaks were observed based on their retention time and ultraviolet (UV) spectrum in all samples (Fig 3A1). All the characteristic chromatograms of 28 baches of samples were shown in S1A and S1B Fig. These 11 flavonoid components were all flavonoid *C*-glycosides and their structures have been identified in our previous research [26]. The similarity evaluation results showed that imitating wild samples had high similarity (from 0.827 to 0.963) while greenhouse cultivation samples exhibited slightly lower similarity (from 0.724 to 0.962), which indicated the quality of *D*. *huoshanense* under imitation wild cultivation mode might be more stable on flavonoids than greenhouse cultivation. In addition, the similarity of contrast chromatograms between two cultivation modes was 0.927. To sum up, whether it was different cultivation modes or different growth years, the characteristic peaks of flavonoid components of *D*. *huoshanense* were basically the same. For another, as peak area heat map shown (Fig 3A2), the relative content of flavonoids had a tendency to accumulate with the increase of growth years. However, there was no significant difference between the two cultivation modes.

At 215 nm, the best wavelength for bibenzyls and lignans detection, 13 common characteristic peaks were marked (Fig 3B1). The characteristic chromatograms of all samples were shown in S1C and S1D Fig. Peaks L1~6 were determined to be lignans and peaks B1~5 were identified as bibenzyls based on their UV spectra [37]. Both compounds have maximum absorption at 215 and 270 in the UV spectrum, but the difference is that the UV spectrum of bibenzyl compounds has a higher shoulder peak at the maximum absorption of 215 nm. Moreover, peaks U1 and U2 with relatively high peak areas existed stably in all samples, but it could not be inferred from the UV spectrum information which compound they belonged to. The similarity of imitating wild samples was from 0.903 to 0.982, while the greenhouse cultivation was from 0.849 to 0.958. Similar to flavonoids, it demonstrated that the quality of imitating wild *D*. *huoshanense* also might be more stable on lignans and bibenzyls components. But differently, the peak areas of W-samples’ chromatographic peaks were obviously higher than G-samples, especially peaks L5 and B3, the two most abundant peaks, which indicated that different growing environment could greatly affect the accumulation of lignans and bibenzyls (Fig 3B2). Such a difference also existed among the samples with different growth years. It was worth noting that peaks L1, L5, and L6 were determined to be syringaresinol-4,4-O-β-D-di-glucopyranoside, syringaresinol-4-O-β-D-glucopyranoside, and syringaresinol, respectively through the comparison of retention time and UV absorbance spectrum of reference substance (S2 Fig). These three components have been confirmed to exist in *D*. *huoshanense* previously [38]. In this study, they were isolated stably through HPLC for the first time, which can be used as potential quality indicator components for determination content of *D*. *huoshanense*.

In addition, the content of two flavonoids (schaftoside and isoschaftoside) and three lignans (syringaresinol-4,4-O-β-D-di-glucopyranoside, syringaresinol-4-O-β-D-glucopyranoside, and syringaresinol) were determined under the same conditions as the characteristic chromatogram (Table 3). By comparing the amounts, it was found that the compounds of *D*. *huoshanense* from different cultivation mode were quite different in lignans, but not in flavonoids (Detailed results were shown in S1 Table).

### Volatile compounds and monobenzyls composition

Nowadays, GC and GC-IMS are conventionally used to determine qualitative and quantitative compositions of volatile compounds with various external or internal standard calibration procedures and GC-MS is one of the most widely used methods for analyzing the emissions of biogenic volatile organic compounds (VOCs) from plants [39]. Moreover, the VOCs of natural plants contain a significant number of compounds. They can serve as important substances involved in the Maillard reaction of food [40]. However, researches about VOCs in *Dendrobium* are mainly concentrated on their flowers rather than stems [41]. Up to now, there are few studies on the VOCs of *D*. *huoshanense*.

**Fig 4:**
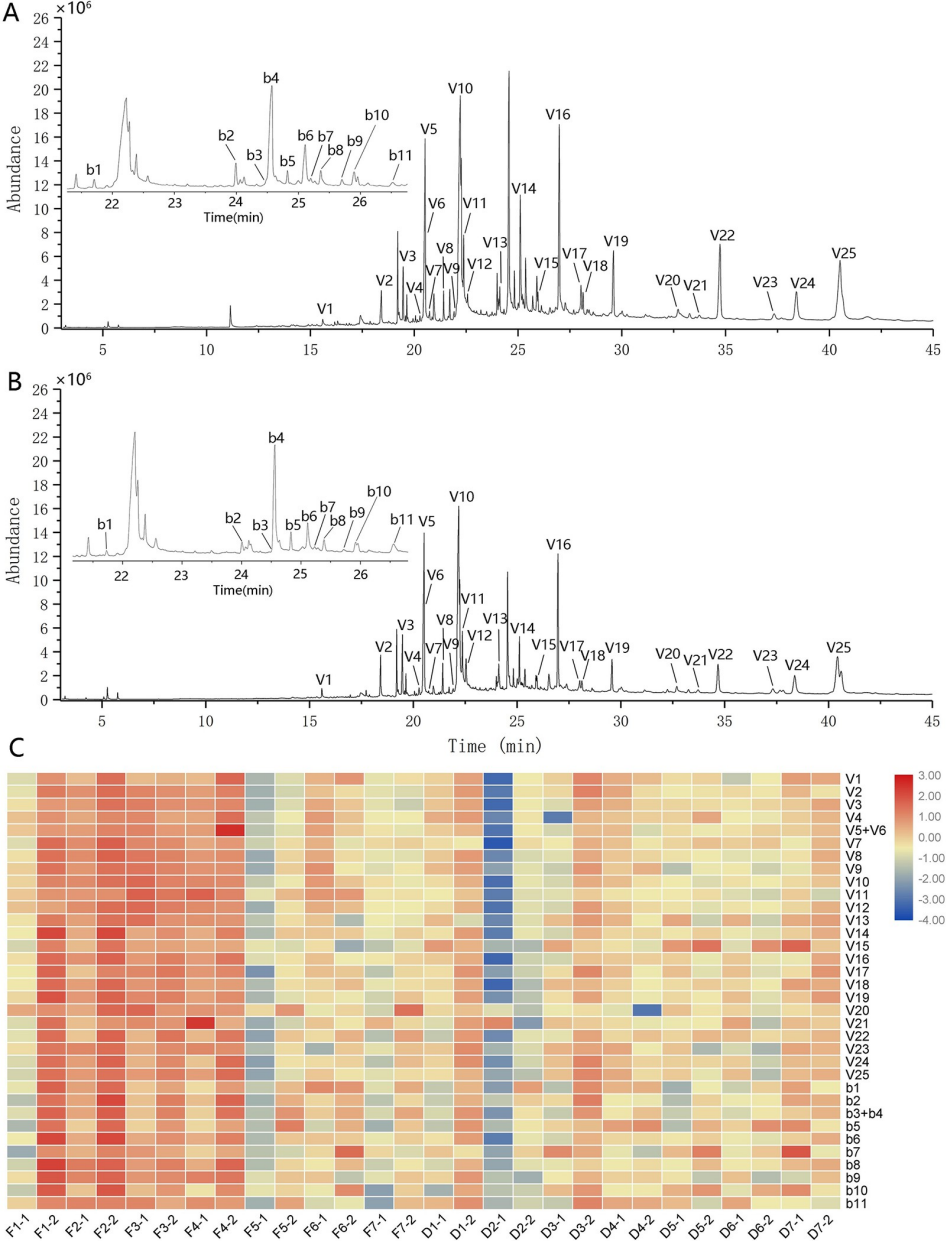
TIC diagrams by GC-MS the identified common peaks of *D*. *huoshanense* under wild imitating (**A**) and greenhouse (**B**) cultivation modes and their heat map of peak area (**C**).

In order to investigate whether the VOCs of *D*. *huoshanense* is associated with its cultivation environments and growth years, we analyzed 28 batches of samples by GC-MS. The total ion chromatographic (TIC) diagrams were shown in S3 and S4 Figs. After optimizing the extraction and separation conditions, more than 50 peaks were resolved in the TIC. Among these, 25 principal common volatile compounds (V1~25) were identified in the NIST 11 mass spectrometry database by selecting results with more than 80% match, and the structures of 11 monobenzyl compounds (b1~11) were deduced from the ion fragment information obtained by mass spectrometry in all the samples (Fig 4A and 4B). Among the 25 identified principal volatile compounds, 8 were identified as fatty acids, 6 were alkanes and their derivatives, 4 were alcohols, 3 were phenols, 2 were esters, and 2 were other compounds (Table 4). These compounds could be detected in all samples of *D*. *huoshanense*, but the relative contents varied greatly among samples from different cultivation methods and growth years (Fig 4C), especially the peaks with retention time between 23~27 min. Through the ion fragment analysis of these peaks, we found that the peaks occurred during this period were mostly monobenzyl compounds.

**Table 4:** Identification of main volatile constituents of *D*. *huoshanense* by GC-MS.

Peak No.	RI	RT (min)	Identification	molecular formula	molecular weight
V1	1860	15.59	Phenol, 2,4-bis(1,1-dimethylethyl)-	C_14_H_22_O	206.17
V2	1439	18.42	Tetradecanoic acid	C_14_H_28_O_2_	228.21
V3	1772	19.47	Pentadecanoic acid	C_15_H_30_O_2_	242.22
V4	1881	20.27	Palmitoleic acid	C_16_H_30_O_2_	254.22
V5	1965	20.28	n-Hexadecanoic acid	C_16_H_32_O_2_	256.24
V6	1966	20.53	Dibutyl phthalate	C_16_H_22_O_4_	278.15
V7	1993	20.74	Hexadecanoic acid, ethyl ester	C_18_H_36_O_2_	284.27
V8	2016	21.42	Heptadecanoic acid	C_17_H_34_O_2_	270.26
V9	2093	21.92	Phytol	C_20_H_40_O	296.31
V10	2156	22.23	9,12-Octadecadienoic acid (Z,Z)-	C_18_H_32_O_2_	280.24
V11	2195	22.28	9,12,15-Octadecatrienoic acid (Z,Z,Z)-	C_18_H_30_O_2_	278.23
V12	2201	22.39	Octadecanoic acid	C_18_H_36_O_2_	284.27
V13	2213	24.13	9-Octadecenamide (Z)-	C_18_H_35_NO	281.48
V14	2405	25.11	Pentacosane	C_25_H_52_	352.68
V15	2500	25.96	Hexacosane	C_26_H_54_	366.71
V16	2600	26.99	Heptacosane	C_27_H_56_	380.73
V17	2700	28.03	13-docosenamide (Z)-	C_22_H_43_NO	337.58
V18	2791	28.13	Octacosane	C_28_H_58_	394.45
V19	2800	29.59	Nonacosane	C_29_H_60_	408.79
V20	2900	32.70	γ-Tocopherol	C_28_H_48_O_2_	416.37
V21	3152	33.73	Stigmastan-3,5-diene	C_29_H_48_	396.38
V22	3247	34.72	Vitamin E	C_29_H_50_O_2_	430.38
V23	3337	37.33	Campesterol	C_28_H_48_O	400.68
V24	3569	38.39	Stigmasterol	C_14_H_22_O	412.37
V25	3666	40.48	γ-Sitosterol	C_14_H_28_O_2_	414.39

RT = retension times; RI = retention indices

**Fig 5:**
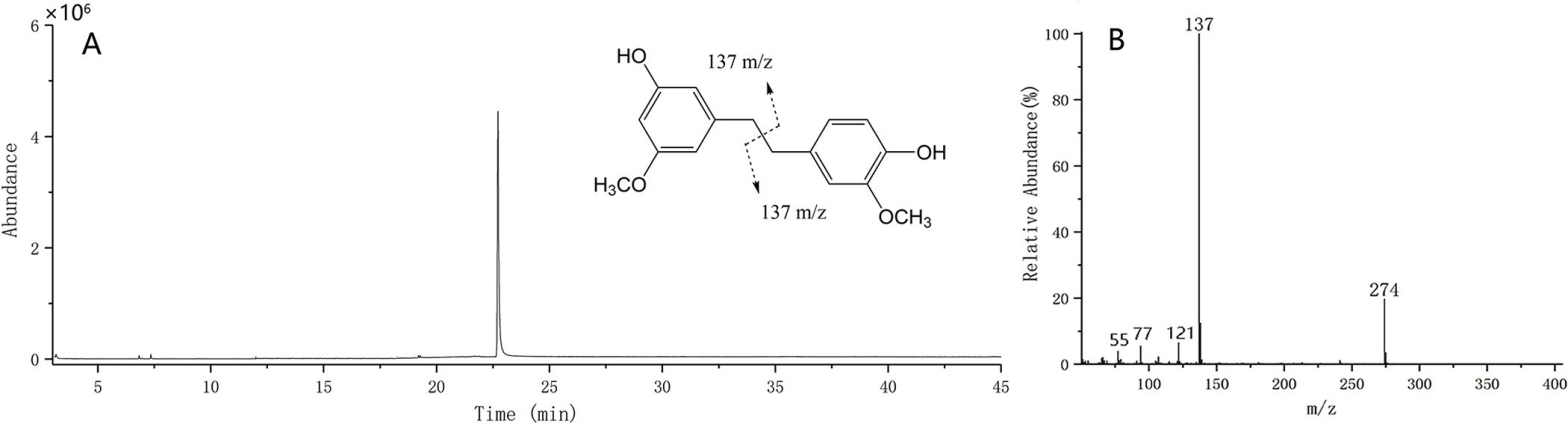
TIC diagram (**A**) and ion fragment diagram (**B**) of reference Gigantol.

Monobenzyl compounds generally produced molecular ion peaks in the EI-MS spectrum and had two strong fragment ion peaks of benzyl cleavage, which can be used to preliminarily infer the type and number of substituents on the two benzene rings [42]. Such as, if there is no substituent on the benzene ring, there will be a strong ion peak of m/z 91, and the strong ion peak of m/z 107 represents a hydroxyl group on the benzene ring; when there is a methoxyl group on the benzene ring, there will be a strong ion peak of m/z 121; if there is a hydroxyl group and a methoxy group, there will be a strong ion peak of 137, and so on. For instance, the reference substance Gigantol was analyzed with this chromatographic condition. As shown in Fig 5, its molecular ion peak is m/z 274, and mainly produced a strong ion peak of m/z 137 after the bridge bond break.

**Fig 6:**
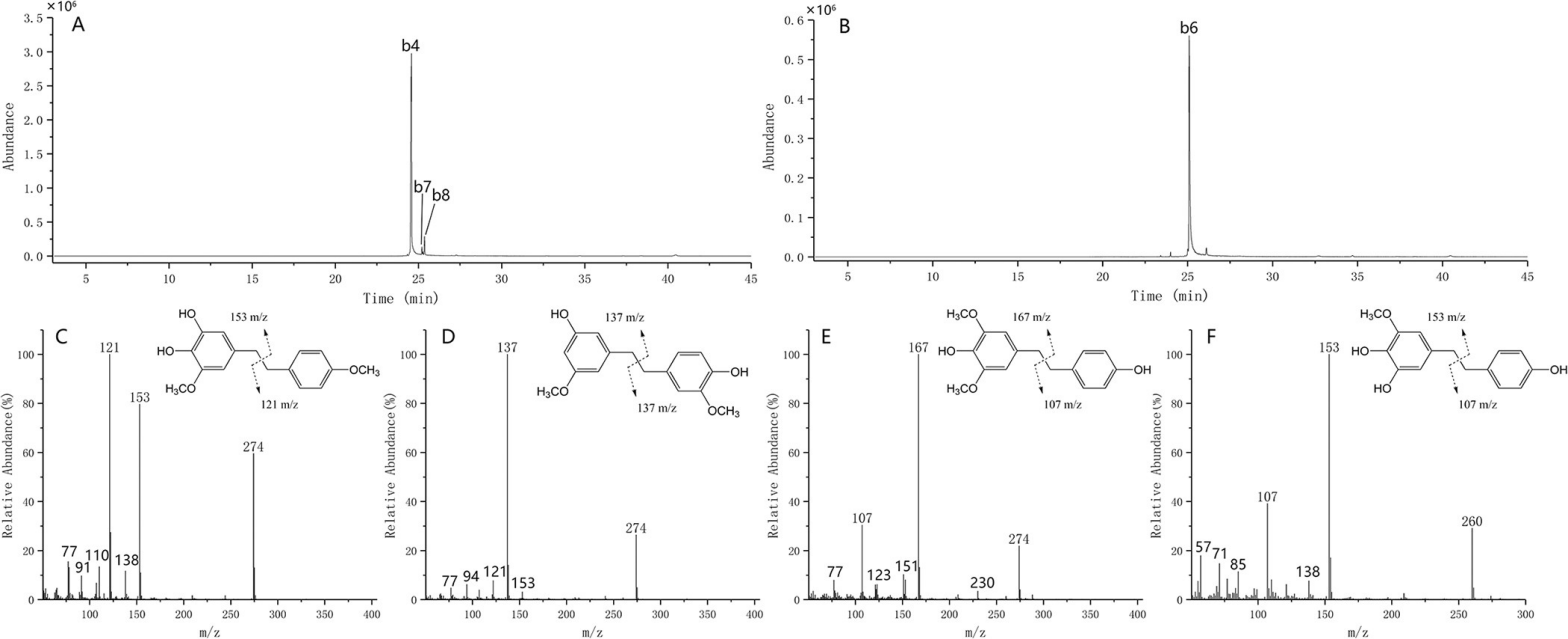
MS diagrams of extraction ions of 274 m/z (**A**), 260 m/z (**B**) and their ion fragmentation diagrams (**C, D** for 274 m/z and F for 260 m/z).

According to the fragmentation regularity of bibenzyl in EI-MS and related literature, a total of 11 monobenzyl components were identified from *D*. *huoshanense*. Identification information is summarized in Table 5. The mass chromatogram of some compound with specific mass could be obtained by selecting the ions from TIC, which enable the target peak to be isolated and remove the interference for further analysis. When the extraction ion is m/z 228, three main chromatographic peaks can be obtained, among which only one (peak b3) conforms to the fragmentation regularity of bibenzyl. The fragment ions at m/z 137 and m/z 91 with relatively high abundance means one benzene ring has a hydroxyl group and a methoxy group attached to it, and the other benzene ring has no substituent. Then peak b1 was identified as 3-hydroxyl-5-methoxy-bibenzyl in combination with reference [43]. As for peak b2, the molecular ion at m/z 258 and the fragment ions at m/z 121 and suggested it was 3-hydroxyl-5,4’-dimethoxy-bibenzyl [43]. The mass spectrum of peak b3 with molecular ion at m/z 244 displayed major fragment ions at m/z 137 and m/z 107. Therefore, by comparison with the reference peak b3 was assigned as 3,4’-dihydroxyl-5-methoxy-bibenzyl [44–46]. There are three molecular ion peaks of m/z 274 in the extraction ion chromatogram, but the characteristic ion fragments of them are not exactly the same (Fig 6A and 6C–6E). Ion fragments at m/z 121 and m/z 153 were produced for peak b4, ions at m/z 167 and m/z 107 were observed for peak b8, whereas ions at m/z 137 only showed for peak b7. Together with these characteristic ions and reference information, peaks b4, b7, and b8 were identified as 3,4-dihydroxyl-5,4’-dimethoxy-bibenzyl, Gigantol, and 4,4’-dihydroxyl-3,5-dimethoxy-bibenzyl, respectively [45, 46]. It should be noted that peak b4 was the highest relative content of all bibenzyl components. Moreover, peak b6 with molecular ion at m/z 260, was the second largest bibenzyl peak. As shown in Fig 6B and 6F, typical ions fragments at m/z 121 and m/z 153 indicated that peak b6 was Dendrosinen B [46]. Peak b5 produced molecular ion at m/z 288, the ion fragments at m/z 167 with high abundance indicated a hydroxyl group and two methoxy groups attached to bibenzyl ring. Combined with that peak b5 has a molecular weight of 14 more than b4, a methyl group. Finally, peak b5 was inferred as 3-hydroxyl-3,5,4’-trimethoxy-bibenzyl. Similarly, peak b10 with molecular ion at m/z 304, which generated ion fragments at m/z 167 and m/z 137, was assigned as Moscatilin [46]. For peak b9 with a molecular ion at m/z 290, the intensity was 30 amu (a methoxy group) higher than that of peak b6. Together with the fragment ions at m/z 137 and m/z 153 in the MS spectra, peak b9 were deduced to be 4,5,4’-trihydroxyl-3,3’-dimethoxy-bibenzyl. Peak b11 were 14 amu than peak b10, combined with the existence of its characteristic ions at m/z 151 and m/z 167, we could tentatively characterise peak 11 as 4’-hydroxyl-3,4,5,3’-tetramethoxy-bibenzyl. In conclusion, monobenzyl compounds generally produced α-α’ cleavage, resulting in molecular ion and two characteristic fragment ions, which could tentatively elucidate the number of hydroxyl and methoxy substituents on the phenyl rings.

**Table 5:** Identification of monobenzyl components and information of ion fragments of *D*. *huoshanense* by GC-MS.

Peak No.	Retention Time (min)	molecular weight	Identification	Ion fragments (relative abundance %)
b1	21.71	228	3-hydroxyl-5-methoxy-bibenzyl	137(100), 228(61), 91(48), 229(10), 77(8), 107(3)
b2	24.00	258	3-hydroxyl-5,4’-dimethoxy-bibenzyl	121(100), 258(22), 77(7), 91(4), 137(2), 107(1)
b3	24.49	244	3,4’-dihydroxyl-5-methoxy-bibenzyl	137(100), 244(54), 107(44), 77(16), 121(9), 153(5), 91(5)
b4	24.57	274	3,4-dihydroxyl-5,4’-dimethoxy-bibenzyl	121(100), 153(80), 274(60), 77(16), 91(10), 107(7)
b5	24.83	288	3-hydroxyl-3,5,4’-trimethoxy-bibenzyl	167(100), 121(68), 288(26), 168(11), 153(9), 77(7), 123(5), 91(5), 274(4), 107(3)
b6	25.11	260	Dendrosinen B	153(100), 107(39), 260(29), 57(18), 154(17), 71(15), 85(11), 77(8), 121(6)
b7	25.20	274	Gigantol	137(100), 274(26), 138(14), 122(8), 94(6), 77(5), 107(4),153(3), 121(2), 55(2)
b8	25.36	274	4,4’-dihydroxyl-3,5-dimethoxy-bibenzyl	167(100), 107(30), 274(22), 151(10), 153(8), 77(8), 121(6), 130(3), 81(3)
b9	25.71	290	4,5,4’-trihydroxyl-3,3’-dimethoxy-bibenzyl	137(100), 153(83), 290(32),55(23), 121(23), 73(21), 57(21), 71(15), 129(15), 107(14)
b10	25.90	304	Moscatilin	167(100), 151(67), 137(39), 304(38), 153(22), 107(7), 121(7), 123(6), 77(5), 81(4)
b11	26.12	318	4’-hydroxyl-3,4,5,3’-tetramethoxy-bibenzyl	151(100), 167(76), 137(39), 242(35), 121(27), 153(27), 318(25), 55(21), 152(20), 107(20), 69(16), 77(12), 123(11), 260(9)

### Multivariate statistical analysis of PCA, OPLS-DA and VIP

**Fig 7:**
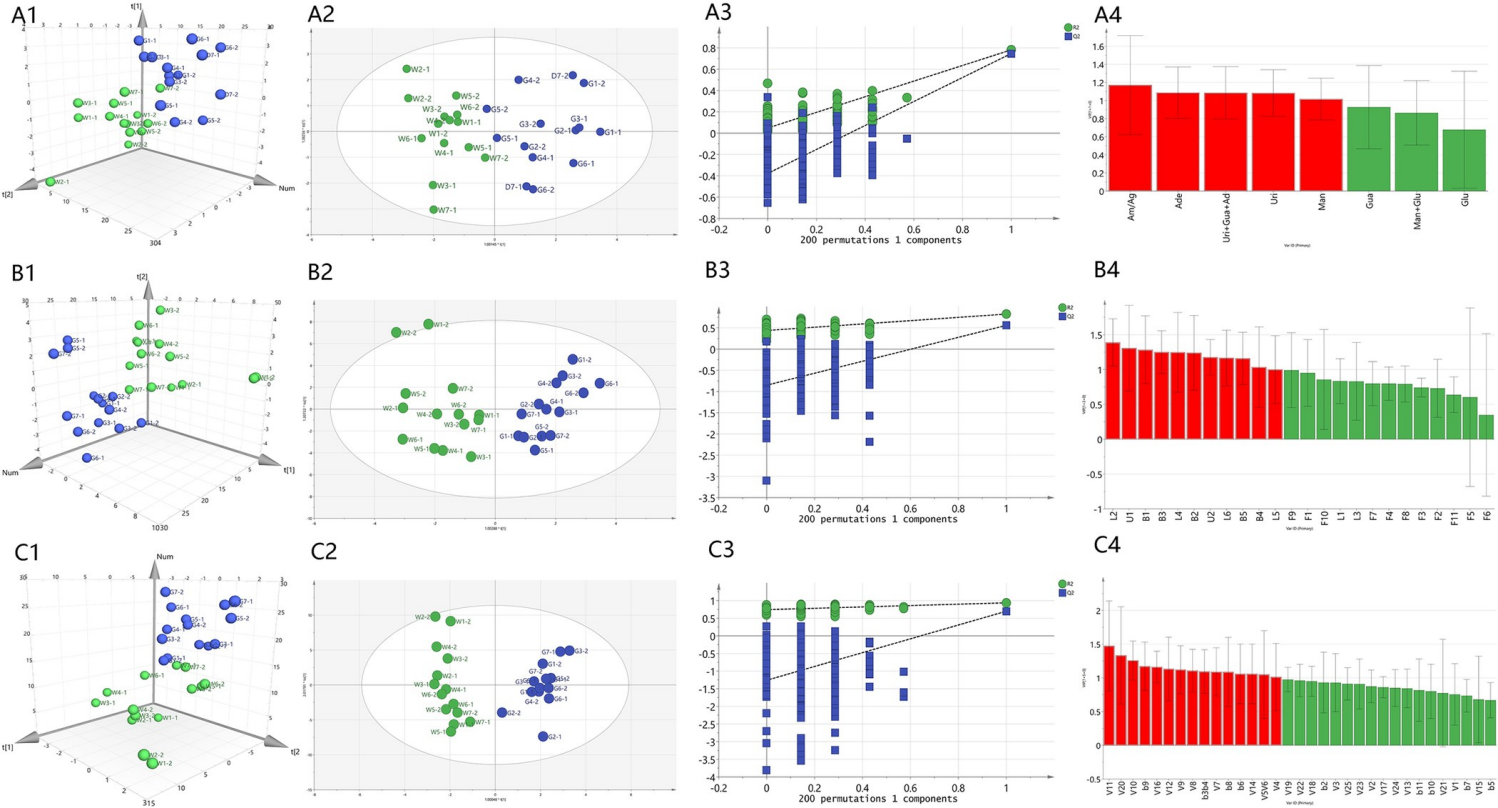
PCA, OPLS-DA and VIP analysis of multi-components in *D*. *huoshanense* under wild imitating and greenhouse cultivation modes. **A1-A4:** PCA, OPLS-DA, permutation test and VIP analysis of polysaccharides and nucleosides; **B1-B4:** PCA, OPLS-DA, permutation test and VIP analysis of flavonoids, bibenzyls and lignans; **C1-C4:** PCA, OPLS-DA, permutation test and VIP analysis of mobobenzyls and VOCs.

Multivariate statistical analysis was used to distinguish *D*. *huoshanense* between imitation wild and greenhouse cultivation. We conducted PCA analysis based on polysaccharides content, monosaccharide ratio and nucleosides content, the peak areas of 24 flavonoids, bibenzyls, lignans from HPLC, and the peak areas of 36 monobenzyls, VOCs from GC-MS, respectively. As shown in Fig 7A1–7C1, these components could distinguish the two kinds of cultivation mode, The plots of the two PCs (PC1 and PC2) of these three PCA models demonstrated R^2^X and Q^2^ values of over 65%. The PCA score plots exhibited high goodness of fit (R^2^X = 0.854, 0.660 and 0.796, respectively) and predictability (Q^2^ = 0.687, 0.693 and 0.714, respectively)-greater than 0.5 for each model. To find significant differences between the two groups of related chemical components, the same data were subject to OPLS-DA analysis (Fig 7A2–7C2). In this model, their R^2^X, R^2^Y, and Q^2^ values were (R^2^X = 0.713, R^2^Y = 0.771, Q^2^ = 0.719) in polysaccharides and nucleosides, (R^2^X = 0.778, R^2^Y = 0.824, Q^2^ = 0.558) in flavonoids and lignans, (R^2^X = 0.887, R^2^Y = 0.937, Q^2^ = 0.699) in monobenzyls and VOCs, respectively, with the good fit and predictability. And the results of permutation test (200 times) showed that the intercept of regression line of Q^2^ is less than 0, which indicated that there was no overfitting of the model and the model validation is effective (Fig 7A3–7C3). Further analysis ranked the importance of these compounds to separation into groups (Fig 7A4–7C4). Those compounds with a variable importance in projection (VIP) score of ≥1 were considered to significantly contribute to separation and serve as candidate markers for the cultivation mode. As the results showed, the most influential components to distinguish the two cultivation modes were monosaccharide ratio, adenosine, total nucleosides, uridine and mannose content, the bibenzyls of B1, B3, B2, B5, B4, lignans of L2, L4, L6, L5 and unknown compounds U1, VOCs of V11, V10, V16, V12, V9, V8, V7, V14, V5, V6, V4 and monobenzyls of b9, b3, b4, b8, b6.

### Comprehensive comparative analysis on multiple chemical compositions

The difference between different cultivation modes and growth years of *D*. *huoshanense* on the main components were comprehensively analyzed based on content or peak area from HPLC and GC-MS analysis.

**Fig 8:**
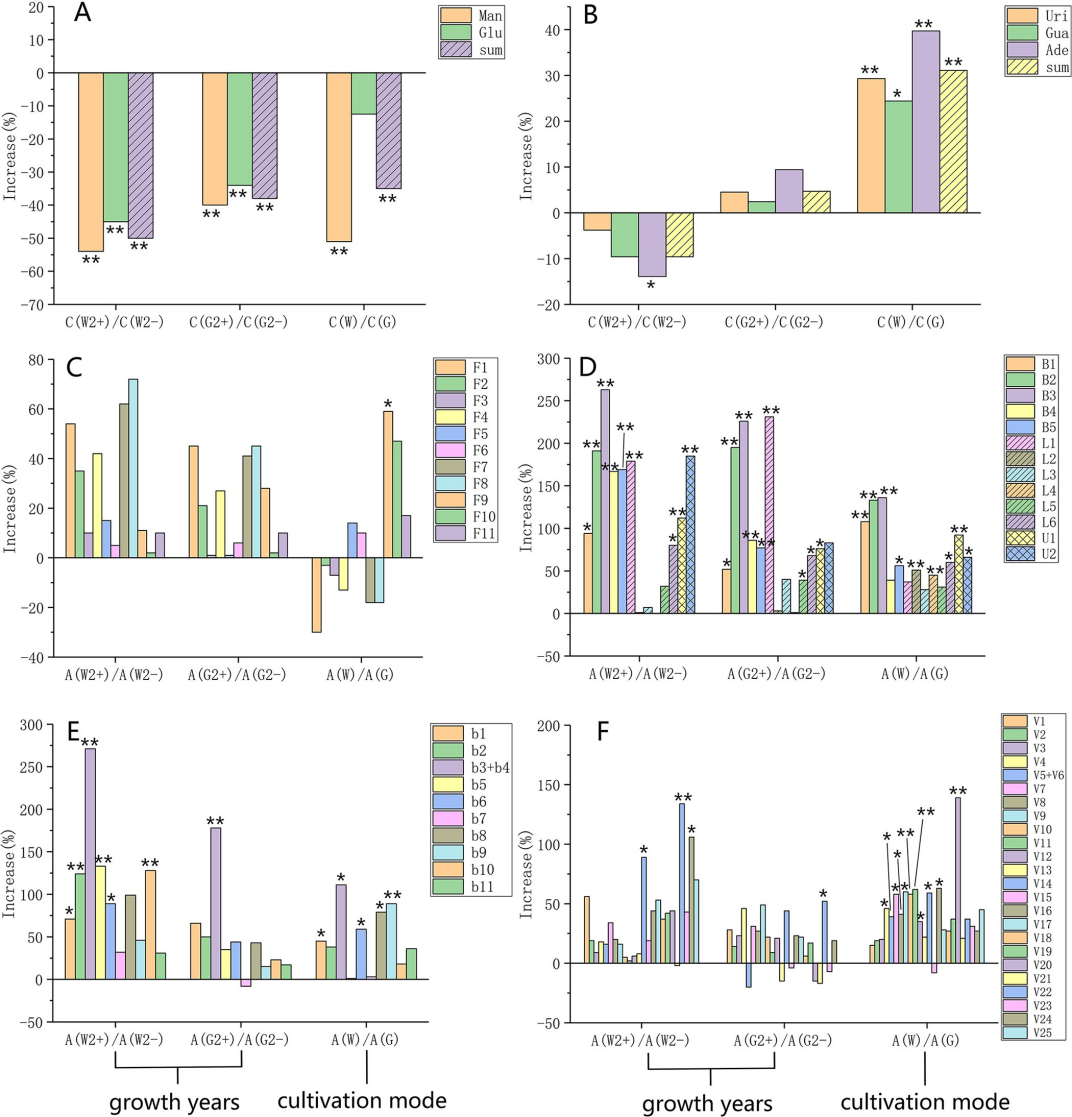
Comprehensive comparison analysis of main components in *D*. *huoshanense* between two different growth years and two different cultivation modes (**A:** polysaccharides by content determination; **B:** nucleosides by content determination; **C:** flavonoids by HPLC; **D:** bibenzyls and lignans by HPLC; **E:** monobenzyls by GC-MS; **F:** volatile components by GC-MS. (W: wild imitating cultivation; G: greenhouse cultivation; 2^-^ means less than 2-year-old; 2^+^ means more than 2-year-old; *means P < 0.05; ** means P < 0.01).

*Polysaccharides*. As shown in Fig 8A, the content of monosaccharides and total polysaccharides of *D*. *huoshanense* under two cultivation modes were both significantly decreased (P<0.01) with the increase of growth years. Wild imitating cultivation reduced by about 50%, while greenhouse cultivation reduced by about 40%, and the former was reduced by more. It is reasonable because plants need to consume polysaccharides for energy as they grow. In addition, the content of polysaccharides in imitation wild was 40% lower than that in greenhouse. Except for glucose, the content of mannose and total polysaccharides had very significant difference (P<0.01). Contrary to previous findings, which founded the total polysaccharides content of *D*. *huoshanense* planted in stone under the forest were higher than those planted in greenhouse [25], our study showed that greenhouse cultivation contains more polysaccharides. It is supposed that this may be related to harvest time, or the fact that the polysaccharides of plants will be transformed into glycoside components to resist environmental stress when they growing closer to the wild.

*Nucleotides*. As Fig 8B shown, content of uridine, guanosine and adenosine nucleotides and total nucleotides content of imitation wild cultivated *D*. *huoshanense* were both weakly decreased with the increase of growth years, while the greenhouse showed opposite trend. Notably, the content of nucleotides of imitation wild was about 30% higher than the greenhouse cultivation (P<0.01 or P<0.05). It has been reported that nucleotide metabolism was potentially contribute to the drought tolerance of Dendrobium, which may be the reason for the higher content in the imitation wild samples [47].

*Flavonoids*. As opposed to polysaccharides, the relative contents of flavonoids of *D*. *huoshanense* with more than 2-year-old were higher than that of less than 2-year-old, whether it was imitation wild or greenhouse cultivation. Among them, the content of Among them, the content of compounds F8 (Apigenin-6-C-α-L-arabinoside-8-C-α-L-rhamnosiyl-(1→2)-β-D-glucoside), F7 (Apigenin-6-C-α-L-rhamnosyl-(1→2)-β-D-glucoside-8-C-α-L-arabinoside), F1 (Vitexin Ⅱ) increased the most with the years (increased 40%~70%), but the difference between all flavonoids was not significant (Fig 8C). The content of compounds F5, F6, F9, F10, F11 in imitation wild were higher but the other compounds were all lower than that of greenhouse. Interestingly, the compounds F9 (Apigenin-6,8-di-C-α-L-arabinoside), which was the most abundant component in *D*. *huoshanense*, showed significant difference between the two different cultivation modes (P<0.05, imitation wild was 60% higher than greenhouse). In a word, cultivation mode had little effect on the flavonoid components. Previous study reported that the polyphenolic flavonoid of the majority of the cultivated herbs was no lower than that of their wild relatives [48]. Thus, this showed that greenhouse cultivation was beneficial to the biosynthesis of flavonoids of *D*. *huoshanense* and it were suitable for cultivation.

*Bibenzyls and lignans*. Obviously, as shown in Fig 8D, growth year has a great influence on the accumulation of bibenzyls and lignans in *D*. *huoshanense*. Many components of more than 2-year-old have increased by 50% or even up to 250% compared to those less than 2-year-old. And the relative contents of the two unknown components U1 and U2 are also greatly increased by 50% with the growth years, especially in the imitation wild samples. Most of these components have significant differences (P<0.05 or P<0.01), especially compounds B2, B3, and L1 (syringaresinol-4,4-O-β-D-di-glucopyranoside). On the other hand, the imitation wild *D*. *huoshanense* had more abundant bibenzyls and lignans with 25%~125% higher than greenhouse cultivation. Interestingly, the relative content of monobenzyls identified by GC-MS analysis (Fig 8E) was also higher in more than 2-year-old samples and imitating wild cultivated samples, which was consistent with the result of the bibenzyls by HPLC above.

*VOCs*. We also conducted the comparative analyses of relative content of volatile components based on the peak area by GC-MS (Fig 8F). The results suggested that most of the volatile components increased with the growth years, especially in imitation wild *D*. *huoshanense*. In these ingredients, only compounds V5+V6 (n-Hexadecanoic acid + Dibutyl phthalate), V14 (Pentacosane), V16 (Heptacosane) had significant differences (P<0.05). And most VOCs in imitation wild samples were over 25% higher than that of greenhouse, which was consistent with the previous study that wild samples had more abundant VOCs [49]. This was rational to explain why wild *D*. *huoshanense* was more aromatic. Furthermore, plants synthesize and release a large number of VOCs, which play important roles in their interactions with the environment, such as attracting pollinators and seed dispersers and acting as protective agents such as insect repellents and pathogen inhibitors [50, 51]. *D*. *huoshanense* grown in the wild requires more of these ingredients.

Collectively, on the one hand, the results showed that the cultivation mode had minor impact on flavonoids content, but polysaccharides, nucleosides, bibenzyls, lignans, and volatile compounds were influenced by the mode of cultivation in all tested cultivars. Imitation wild cultivated *D*. *huoshanense* had more abundant nucleosides, bibenzyls, lignans and volatile compounds than that cultivated in greenhouse, but lower polysaccharide content. The most significant differences were in bibenzyls and lignans. On the other hand, growth years have a positive effect on the accumulation of flavonoids, bibenzyls, lignans, volatile compounds in *D*. *huoshanense*, but a negative effect on polysaccharides. Among them, the effect on the composition of bibenzyls were the most significant. Plant secondary metabolites are the important constituents in medicinal plants, the differences between imitation wild and greenhouse cultivated *D*. *huoshanense* might not be the presence or absence of one or more compounds, but rather the content variation. In a word, *D*. *huoshanense* with different cultivation modes and growth years had similar compositions, but they influence and modulate the concentration of compounds in plants differently. In this respect, the evidence showed that the greenhouse cultivation mode was capable of providing an appropriate ambiance for the synthesis of bioactive compounds, regardless of the quality that plants may reach.

One of the main goals of studying the chemistry of natural compounds is to screen bioactive substances from plants. The limitation of our study is that we could not determine the content of some major ingredients due to the absence of reference materials and there are still some components that have not been analyzed. The previous study indicated that living tree epiphytic cultivation was more conducive to the accumulation of active ingredients than forest gap and greenhouse cultivated in *D*. *huoshanense* by comparing the content of polysaccharide, dendrobine and amino acid [52]. There were also studies that analyzed and compared the differences in metabolic components of *D*. *huoshanense* with different growth years, which suggested the optimal harvest time for *D*. *huoshanense* was in the third year [53]. At present, there are few studies on the influence of cultivation methods on the chemical composition of *D*. *huoshanense*. Therefore, our study was detailed and reliable, and more comprehensively compared the differences between the two cultivation methods. Although an imitation wild mode is an effective cultivation mode for the production of compounds, greenhouse mode had their own advantages. The greenhouse cultivation mode proved to be an adequate setting for the production of polysaccharides while the nucleosides, bibenzyls, lignans and VOCs were better synthesized in the imitation wild cultivation mode. Nowadays, “Mountain Delicacies Grow in Wild” idea is more meeting the consumer’s conceptual standard for medicinal plants. The product price of *D*. *huoshanense* under imitating wild cultivation mode is considerably higher (usually about 5–10 times higher) than that in greenhouse cultivation. Our experimental results suggested the reason why imitation wild dendrobium is precious. With regard to productivity, the yield of greenhouse cultivation was higher and it was recommended as functional food or health care products in daily life, while imitation wild *D*. *huoshanense* was more suitable as a drug for clinical treatment. Moreover, even the growth years were less than 2 years old, it also had a rich chemical composition, but the content of some composition was relatively low, which could use as food. However, the molecular mechanisms responsible for these differences are unclear. *D*. *huoshanense* with different cultivation mode and growth years might be used to treat different diseases based on their active ingredients. Further research is required to discover this.

## Conclusions

As medicinal *D*. *huoshanense* are increasingly valued by consumers as a natural and healthy food source, it is necessary to reveal their main components and establish reliable detection methods for qualitative or quantitative analysis. The present study revealed that the kinds of constituents of *D*. *huoshanense* cultivated in greenhouse were basically the same as those in imitation wild, but the content was significantly different in many compounds. Considering all of the factors, *D*. *huoshanense* with imitation wild cultivation have better quality than greenhouse cultivation, and growth years of more than 2-year-old is better that of less than 2-year-old. It is worth mentioning that, the overall quality of wild imitating and greenhouse cultivated *D*. *huoshanense* appeared to be comparable since it has similar ingredients to imitation wild. Even in some cases (e.g., polysaccharides) cultivated plants showed better results than wild plants, which showed that greenhouse cultivation is a successful and sustainable method. Our findings contribute new insights to discriminate and comprehensively evaluate *D*. *huoshanense* of two different cultivation mode and provide a good reference for investigating its nutritional and medicinal value.

## Supporting information

S1 Fig Characteristic chromatogram and contrast chromatogram at 340 nm (**A & B**) and 215 nm (**C & D**) of *D*. *huoshanense* with imitating wild and greenhouse cultivation modes. (S1~S14 represents samples W1-1~W7-2 or G1-1~G7-2; R: contrast chromatogram).
(TIF)

S2 Fig The HPLC chromatograms of *D*. *huoshanense* sample W2-2 (S) and mixed standards (R) and the UV spectra of three standards: L1, L5 and L6. (TIF)

S3 Fig TIC diagrams of 14 batches of *D*. *huoshanense* with imitating cultivation modes by GC-MS. (TIF)

S4 Fig TIC diagrams of 14 batches of *D*. *huoshanense* with greenhouse cultivation modes by GC-MS. (TIF)

S1 Table Contents of 10 compounds and monosaccharide ratio of wild imitating (W) and greenhouse cultivated (G) *D*. *huoshannense* (mean±SD, n = 2). TPC: Total polysaccharide content; Am/Ag: monosaccharide ratio.
(DOCX)
