# Supplementary figures and images for: Wild imitating vs greenhouse cultivated Dendrobium huoshanense: Chemical quality differences
Source: PLoS One. 2024 Jan 25;19(1):e0291376. doi: 10.1371/journal.pone.0291376 (PMC10810538; doi:10.1371/journal.pone.0291376)

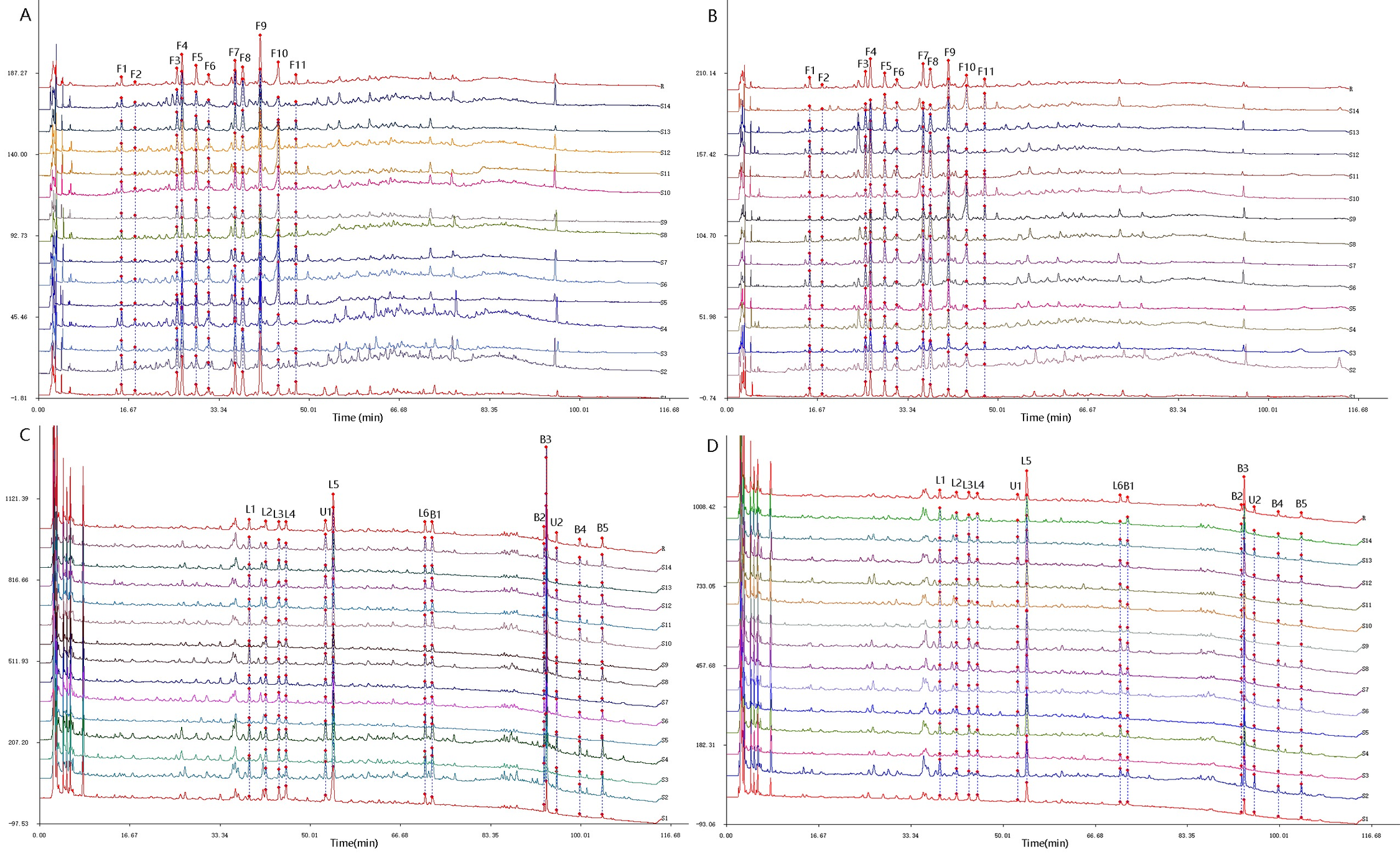

Supplement: S1 Fig — Characteristic chromatogram and contrast chromatogram at 340 nm (A & B) and 215 nm (C & D) of D. huoshanense with imitating wild and greenhouse cultivation modes. (S1~S14 represents samples W1-1~W7-2 or G1-1~G7-2; R: contrast chromatogram). (TIF) [file pone.0291376.s001.tif]

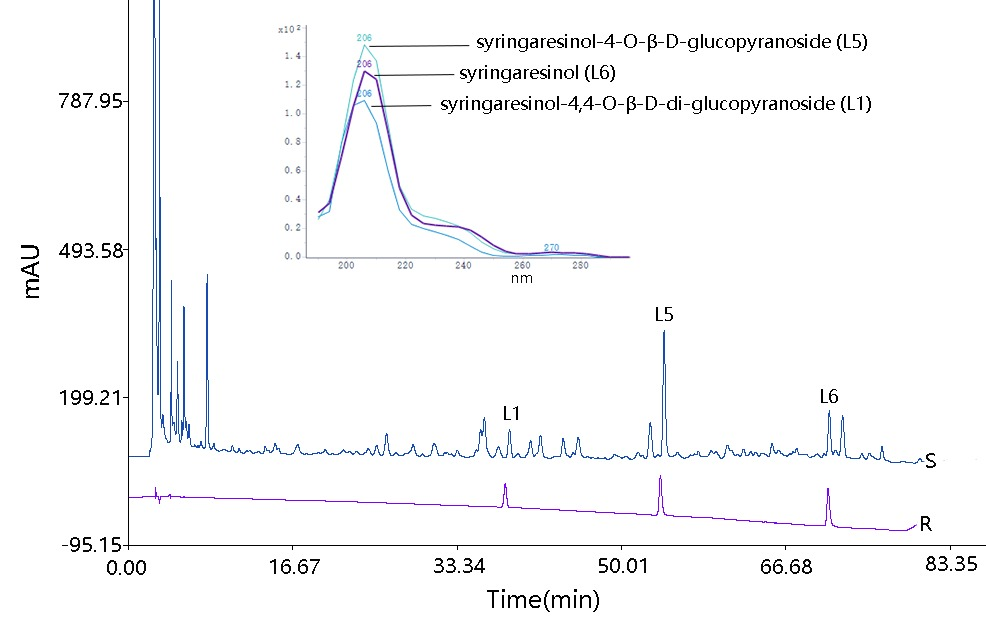

Supplement: S2 Fig — (TIF) [file pone.0291376.s002.tif]

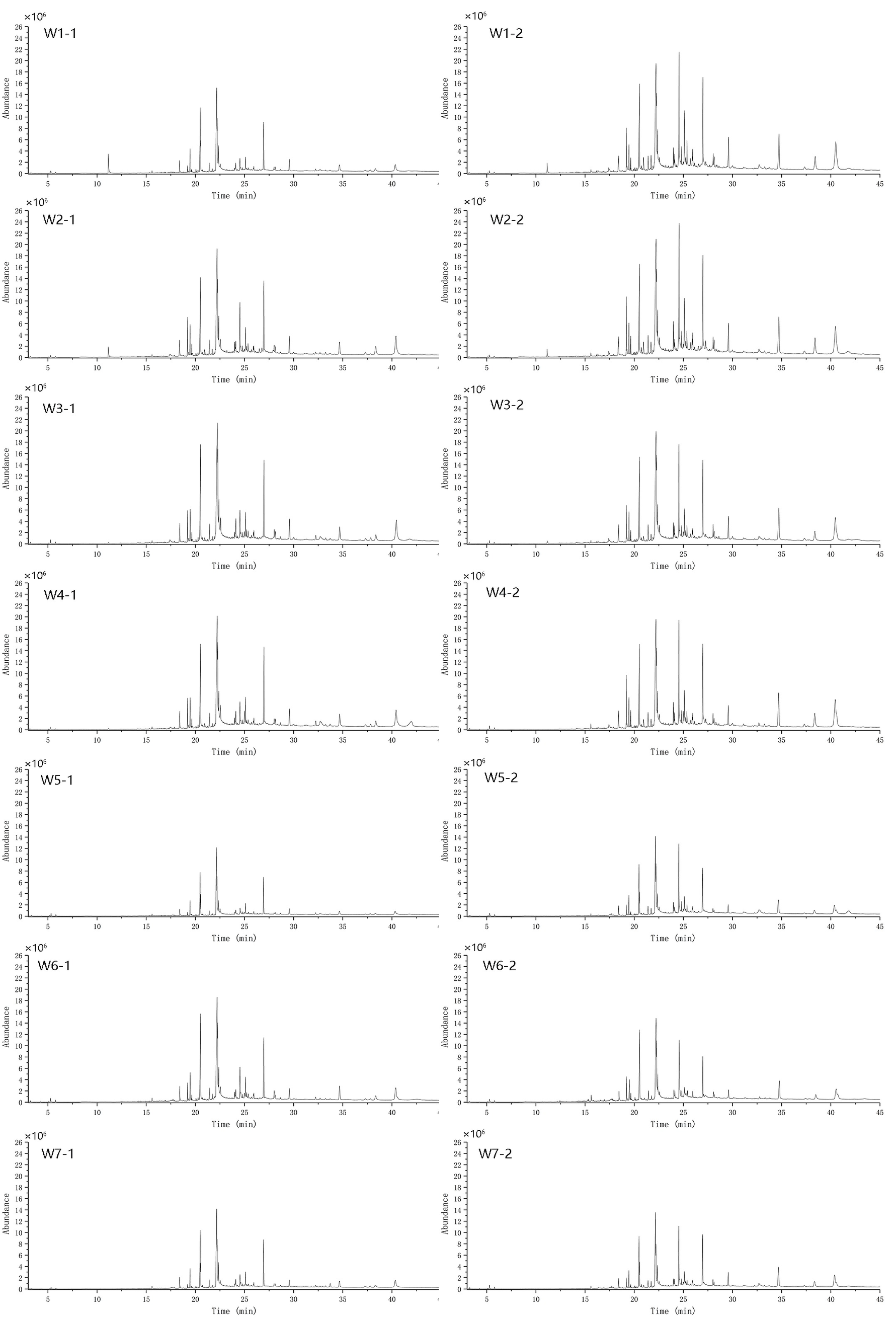

Supplement: S3 Fig — (TIF) [file pone.0291376.s003.tif]

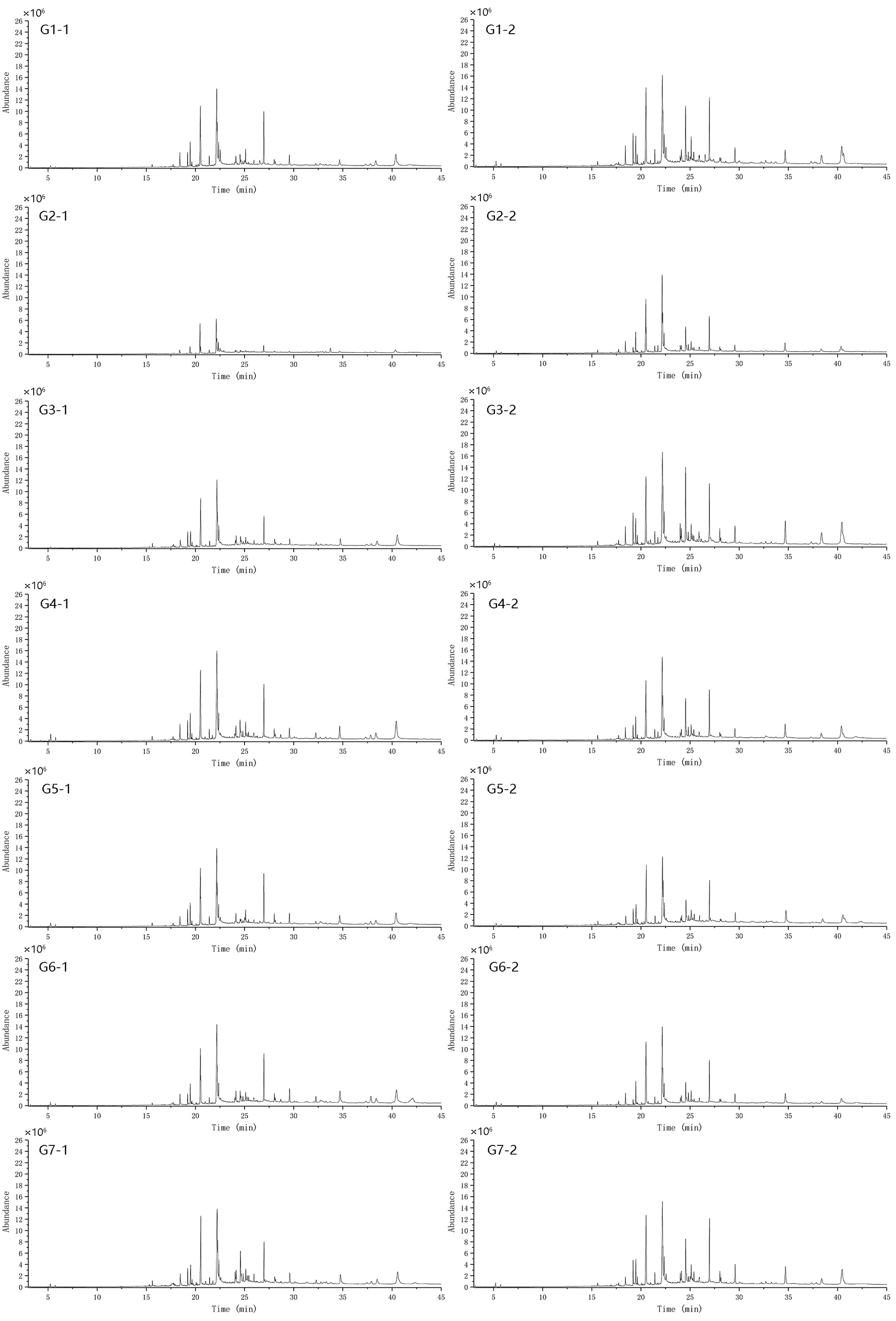

Supplement: S4 Fig — (TIF) [file pone.0291376.s004.tif]
