# Supplementary material for: Wild imitating vs greenhouse cultivated Dendrobium huoshanense: Chemical quality differences
Source: PLoS One. 2024 Jan 25;19(1):e0291376. doi: 10.1371/journal.pone.0291376 (PMC10810538; doi:10.1371/journal.pone.0291376)
Supplement: S1 Table — TPC: Total polysaccharide content; Am/Ag: monosaccharide ratio. (DOCX) [file pone.0291376.s005.docx]

**Table S1** Contents of 10 compounds and monosaccharide ratio of wild imitating (W) and greenhouse cultivated (G) *D. huoshannense* (mean±SD, n=2)

| Samples | Polysaccharide Content (%) | | | | Nucleoside Content (μg/g) | | | Flavonoid Content (μg/g) | | Lignans Content (μg/g) | | |
| --- | --- | --- | --- | --- | --- | --- | --- | --- | --- | --- | --- | --- |
|  | D-mannose | D-glucose | TPC | Am/Ag | Uridine | Guanosine | Adenosine | Schaftoside | Isoschaftoside | Syringaresinol-di-glucopyranoside | Syringaresinol-  glucopyranoside | Syringaresinol |
| W1-1 | 12.68±0.17 | 12.78±0.29 | 25.45±0.46 | 1.07±0.01 | 222.63±1.02 | 261.88±0.69 | 262.25±0.23 | 87.63±2.01 | 133.86±2.97 | 86.53±0.65 | 400.56±5.39 | 68.21±0.54 |
| W1-2 | 0.69±0.02 | 5.17±0.14 | 5.86±0.12 | 0.15±0.01 | 185.44±4.34 | 203.31±2.43 | 200.03±2.28 | 105.13±2.31 | 206.01±4.28 | 395.00±0.10 | 727.75±1.59 | 137.77±0.09 |
| W2-1 | 11.93±0.06 | 11.97±0.40 | 23.91±0.34 | 1.10±0.03 | 301.83±6.77 | 377.17±8.43 | 341.97±8.20 | 89.49±0.95 | 156.52±5.32 | 109.97±2.69 | 553.15±9.40 | 90.37±2.16 |
| W2-2 | 0.87±0.01 | 4.86±0.02 | 5.73±0.03 | 0.19±0.01 | 239.37±2.02 | 250.87±3.54 | 247.27±3.96 | 94.58±0.29 | 197.95±0.44 | 288.21±0.11 | 655.11±0.88 | 163.21±0.14 |
| W3-1 | 14.54±0.02 | 19.63±0.19 | 34.17±0.17 | 0.80±0.01 | 203.68±1.21 | 240.14±1.43 | 255.29±6.51 | 45.54±0.16 | 41.97±0.15 | 121.41±1.36 | 186.70±0.89 | 34.64±0.24 |
| W3-2 | 5.28±0.03 | 8.35±0.06 | 13.63±0.03 | 0.69±0.01 | 218.97±5.90 | 221.69±5.42 | 238.49±5.91 | 42.86±1.28 | 66.73±2.11 | 272.20±0.08 | 250.12±0.10 | 44.66±0.32 |
| W4-1 | 10.15±0.18 | 12.92±0.34 | 23.07±0.53 | 0.85±0.01 | 201.95±4.50 | 236.92±5.64 | 259.56±5.39 | 44.28±0.27 | 57.22±0.76 | 179.60±0.23 | 292.58±0.03 | 49.01±0.00 |
| W4-2 | 7.34±0.02 | 10.33±0.02 | 17.66±0.00 | 0.77±0.00 | 208.49±3.32 | 247.69±6.10 | 239.43±4.53 | 49.88±0.02 | 81.26±0.58 | 380.93±5.44 | 332.05±0.06 | 82.71±0.07 |
| W5-1 | 13.33±0.43 | 13.73±0.39 | 27.06±0.04 | 1.05±0.05 | 190.30±0.24 | 223.82±3.24 | 247.11±4.51 | 50.93±0.61 | 68.18±1.46 | 146.67±0.29 | 278.31±0.18 | 39.03±0.28 |
| W5-2 | 5.26±0.03 | 6.45±0.04 | 11.71±0.01 | 0.87±0.03 | 217.22±1.41 | 235.41±1.97 | 248.05±1.56 | 57.93±0.04 | 100.20±0.25 | 308.38±0.00 | 319.40±0.09 | 92.20±0.01 |
| W6-1 | 7.14±0.16 | 11.49±0.12 | 18.63±0.28 | 0.67±0.01 | 220.07±1.50 | 233.58±2.50 | 271.54±5.54 | 52.34±1.76 | 65.37±1.20 | 140.08±0.11 | 324.56±0.14 | 47.82±0.04 |
| W6-2 | 6.12±0.11 | 8.70±0.17 | 14.82±0.29 | 0.77±0.00 | 212.16±5.89 | 224.73±0.20 | 226.88±3.67 | 51.02±0.93 | 101.62±2.17 | 220.21±0.11 | 271.11±0.02 | 63.54±0.01 |
| W7-1 | 14.60±0.10 | 17.47±0.11 | 32.07±0.21 | 0.91±0.00 | 171.50±1.99 | 161.57±2.46 | 289.77±1.79 | 70.26±1.93 | 105.53±3.76 | 114.38±0.02 | 472.89±0.03 | 42.73±0.00 |
| W7-2 | 10.56±0.21 | 10.11±0.29 | 20.68±0.50 | 1.13±0.01 | 153.38±0.02 | 145.82±1.10 | 244.46±3.17 | 80.64±1.12 | 150.64±3.70 | 268.82±0.25 | 539.31±0.76 | 113.76±0.07 |
| G1-1 | 25.06±0.02 | 13.19±0.10 | 38.25±0.08 | 2.07±0.01 | 142.60±3.54 | 166.81±3.50 | 175.45±4.50 | 92.20±1.39 | 118.61±3.13 | 99.46±0.52 | 221.73±0.24 | 25.25±0.01 |
| G1-2 | 11.11±0.03 | 6.36±0.07 | 17.48±0.10 | 1.90±0.01 | 154.65±2.37 | 169.91±3.37 | 160.13±1.86 | 69.39±1.85 | 185.57±1.70 | 329.49±0.82 | 311.90±0.17 | 52.36±0.00 |
| G2-1 | 22.85±0.17 | 14.32±0.21 | 37.17±0.38 | 1.73±0.01 | 174.52±3.96 | 191.78±0.81 | 157.02±4.25 | 70.95±0.38 | 103.24±2.88 | 63.89±0.02 | 240.85±0.72 | 52.62±0.06 |
| G2-2 | 14.10±0.09 | 12.84±0.39 | 26.94±0.48 | 1.19±0.02 | 167.17±4.49 | 175.41±5.25 | 168.44±4.56 | 104.15±2.03 | 125.75±3.68 | 237.52±0.14 | 473.08±0.45 | 84.85±0.13 |
| G3-1 | 23.78±0.17 | 13.11±0.27 | 36.89±0.44 | 1.97±0.02 | 152.96±4.39 | 189.17±4.77 | 196.77±4.51 | 91.65±3.06 | 164.59±6.23 | 74.25±0.05 | 321.88±0.14 | 43.76±0.10 |
| G3-2 | 16.67±0.00 | 11.55±0.08 | 28.21±0.08 | 1.57±0.01 | 172.09±3.90 | 192.24±2.21 | 197.45±4.25 | 94.45±2.69 | 169.31±6.28 | 206.05±1.44 | 412.39±0.23 | 76.26±0.19 |
| G4-1 | 19.37±0.07 | 14.71±0.10 | 34.08±0.04 | 1.43±0.01 | 157.86±2.86 | 179.38±4.03 | 196.45±5.36 | 54.70±0.92 | 142.14±5.39 | 128.18±0.26 | 377.46±0.19 | 68.48±0.07 |
| G4-2 | 12.51±0.02 | 8.84±0.08 | 21.35±0.10 | 1.54±0.01 | 211.09±2.05 | 251.56±1.84 | 222.00±0.55 | 21.29±0.41 | 120.86±2.31 | 340.14±0.10 | 450.50±0.10 | 85.31±0.13 |
| G5-1 | 17.67±0.13 | 15.28±0.01 | 32.95±0.13 | 1.26±0.01 | 198.26±2.93 | 243.36±3.64 | 222.80±3.84 | 36.87±0.60 | 31.73±1.07 | 126.90±0.04 | 183.92±0.15 | 21.08±0.04 |
| G5-2 | 7.56±0.17 | 7.50±0.09 | 15.06±0.26 | 1.10±0.01 | 183.45±4.59 | 209.82±4.61 | 229.44±6.01 | 111.85±0.70 | 56.91±1.40 | 264.43±0.24 | 260.32±0.08 | 59.00±0.10 |
| G6-1 | 21.65±0.07 | 16.77±0.10 | 38.42±0.17 | 1.40±0.01 | 146.82±3.74 | 171.87±4.71 | 114.21±3.32 | 80.00±2.67 | 171.21±6.42 | 101.61±0.08 | 326.33±0.11 | 37.81±0.09 |
| G6-2 | 13.83±0.25 | 14.48±0.23 | 28.31±0.48 | 1.02±0.02 | 110.83±1.27 | 126.33±1.48 | 141.29±0.02 | 98.34±0.31 | 149.52±3.80 | 274.75±0.24 | 446.80±0.04 | 55.15±0.15 |
| G7-1 | 18.65±0.10 | 17.24±0.06 | 35.89±0.04 | 1.17±0.01 | 143.92±1.76 | 161.05±2.45 | 165.27±1.48 | 26.35±0.77 | 135.77±0.44 | 78.13±0.10 | 326.34±0.26 | 46.08±0.05 |
| G7-2 | 13.06±0.28 | 6.68±0.01 | 19.74±0.29 | 2.11±0.03 | 162.88±0.10 | 196.46±2.21 | 210.85±1.64 | 92.20±1.39 | 42.75±0.88 | 236.59±0.34 | 294.13±0.30 | 23.04±0.03 |

TPC: Total polysaccharide content; Am/Ag: monosaccharide ratio.
